# Hypoxia Modulates Fibroblastic Architecture, Adhesion and Migration: A Role for HIF-1α in Cofilin Regulation and Cytoplasmic Actin Distribution

**DOI:** 10.1371/journal.pone.0069128

**Published:** 2013-07-18

**Authors:** Melanie Vogler, Sabine Vogel, Sabine Krull, Katja Farhat, Pia Leisering, Susanne Lutz, Christina M. Wuertz, Dörthe M. Katschinski, Anke Zieseniss

**Affiliations:** 1 Institute of Cardiovascular Physiology, University Medical Center, Georg-August-University Goettingen, Goettingen, Germany; 2 Institute of Pharmacology, University Medical Center, Georg-August-University Goettingen, Goettingen, Germany; University of Nebraska Medical Center, United States of America

## Abstract

Cells can adapt to hypoxia by various mechanisms. Yet, hypoxia-induced effects on the cytoskeleton-based cell architecture and functions are largely unknown. Here we present a comprehensive analysis of the architecture and function of L929 fibroblasts under hypoxic conditions (1% O_2_). Cells cultivated in hypoxia showed striking morphological differences as compared to cells cultivated under normoxic conditions (20% O_2_). These changes include an enlargement of cell area and volume, increased numbers of focal contacts and loss of cell polarization. Furthermore the β- and γ-actin distribution is greatly altered. These hypoxic adjustments are associated with enhanced cell spreading and a decline of cell motility in wound closure and single cell motility assays. As the hypoxia-inducible factor-1α (HIF-1α) is stabilised in hypoxia and plays a pivotal role in the transcriptional response to changes in oxygen availability we used an shRNA-approach to examine the role of HIF-1α in cytoskeleton-related architecture and functions. We show that the observed increase in cell area, actin filament rearrangement, decrease of single cell migration in hypoxia and the maintenance of p-cofilin levels is dependent on HIF-1α stabilisation.

## Introduction

Reduced oxygen availability (hypoxia) is necessary for proper embryonic and fetal development for cells and tissues [1], [2]. Cells also have to face hypoxia under pathological conditions like cardiovascular or chronic lung diseases, stroke and cancer. Moreover during wound healing, vascular injury leads to hypoxic tissue areas through loss in perfusion. Under all these circumstances fibroblasts are one of the cell types found within or migrating into the hypoxic environment. They are pivotal to embryogenesis, tissue repair and tissue remodelling. For example, they play a significant role in pathological hypoxic conditions such as myocardial scar formation after infarction [3], intestinal [4] and cutaneous wound healing. Literature shows heterogeneous effects of hypoxia on fibroblasts: Acute hypoxia can enhance human dermal fibroblasts migration and thus play a positive role in early skin wound healing [5]–[7]. Human pulmonary artery adventitial fibroblasts show an increase in migration [8], however, there is also a recent report demonstrating a reduced migration of dermal fibroblasts under hypoxic conditions [9]. These differences in migration are likely attributable to differences in the experimental setup, e.g. the supply of growth factors [7] and the origin of the cells.

The actin cytoskeleton is fundamental to cell locomotion and changes in migration are associated with dynamic cytoskeleton reorganization. Interestingly it has been shown in different cell types that hypoxia influences members of the Rho family of GTPases [10]–[14], which are master regulators of the actin cytoskeleton [15], [16]. Besides cell motility the actin cytoskeleton governs many other cellular activities like cytokinesis, endocytosis, cell adhesion and cell shape [17]–[20]. Even though some studies have investigated fibroblasts under hypoxic conditions none of them have in depth focused on the morphological consequences of hypoxia on fibroblasts and the associated functional effects. Given the importance of fibroblasts in many tissues in normal and pathological conditions we set out to study the hypoxic adjustments of L929 fibroblasts and found striking changes in cell shape, attachment and motility. These changes are partly related to the hypoxic reorganisation of cytoplasmic actins which depends on the stabilisation of the hypoxia-inducible factor-1α (HIF-1α).

## Results

### Hypoxia Changes Cell Morphology and Focal Contact Assembly

**Figure 1:**
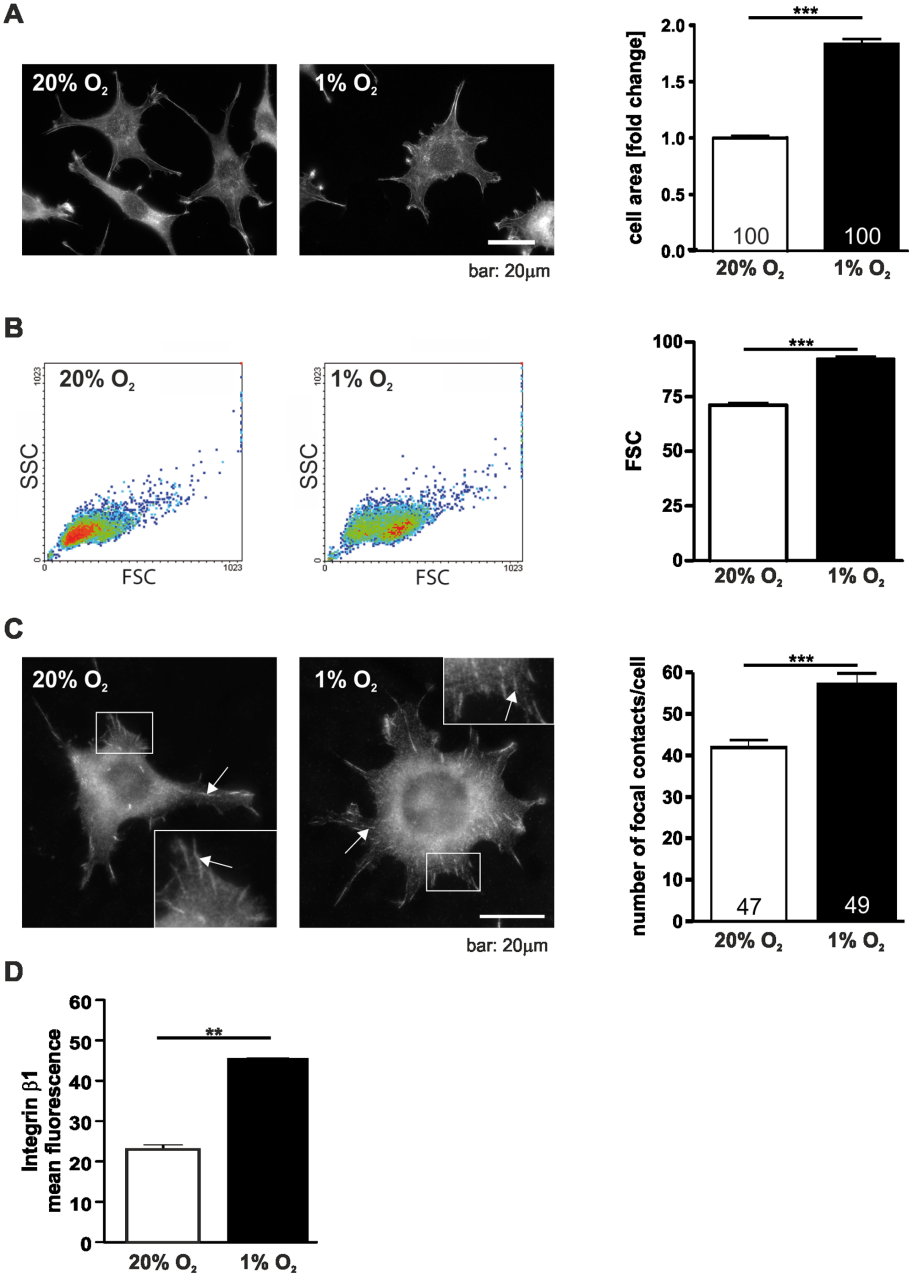
Hypoxia changes cell size and focal contact number. (A) Hypoxia increases the cell area of L929 fibroblasts. Cells were incubated in normoxic (20% O_2_) or hypoxic conditions (1% O_2_) for 24 hrs fixed and stained with phalloidin-FITC. The cell area of single cells was measured and was calculated as fold change compared to 20% O_2._ (B) Flow cytometry analysis of cell volume after incubation in normoxia and hypoxia for 24 hrs. Cells were harvested after 24 hrs. Single cell suspension was prepared by enzymatic digestion. (C) Immunofluorescence images of vinculin in L929 cells. Focal contacts were counted after 24 hrs of hypoxic or normoxic incubation. Note the increase in vinculin positive focal contacts in hypoxia. Arrows point to exemplary focal contacts. (D) Flow cytometry analysis of L929 cells after incubation for 24 hrs in normoxia and hypoxia stained with integrin β1 antibodies. Numbers within the bars indicate the number of cells analysed. ** *p*<0.01, *** *p*<0.001. Bars represent mean values±SEM.

As a first step in investigating the effects of hypoxia on cell architecture L929 fibroblasts were cultivated in normoxic (20% O_2_) and hypoxic (1% O_2_) conditions. Cells cultivated in hypoxia for 24 hrs showed striking morphological changes compared to normoxic control cells (Fig. 1A). Under hypoxic conditions the cell area significantly increased compared to normoxic conditions. To address the question whether the increase in L929 cell area is due to flattening and spreading of the cells or is accompanied by an increase in cell volume the cells were analysed by flow cytometry (Fig. 1B). Measurements of forward-angle light scatter (FSC) showed that the enlarged cell area under hypoxic conditions goes along with a gain in cell volume. To analyse whether this change in cell morphology under hypoxic conditions also correlates with a change in focal adhesions the cells were immunostained for vinculin, a characteristic focal contact protein and focal contacts were quantified (Fig. 1C). 24 hrs of hypoxic incubation led to a significantly increased average number of vinculin positive focal contacts. In line with this result we also observed the accumulation of β1-integrin at the cell surface using flow cytometry (Fig. 1D).

### Advanced Early Cell Spreading and Delayed Cell Migration of L929 Cells in Hypoxia

**Figure 2:**
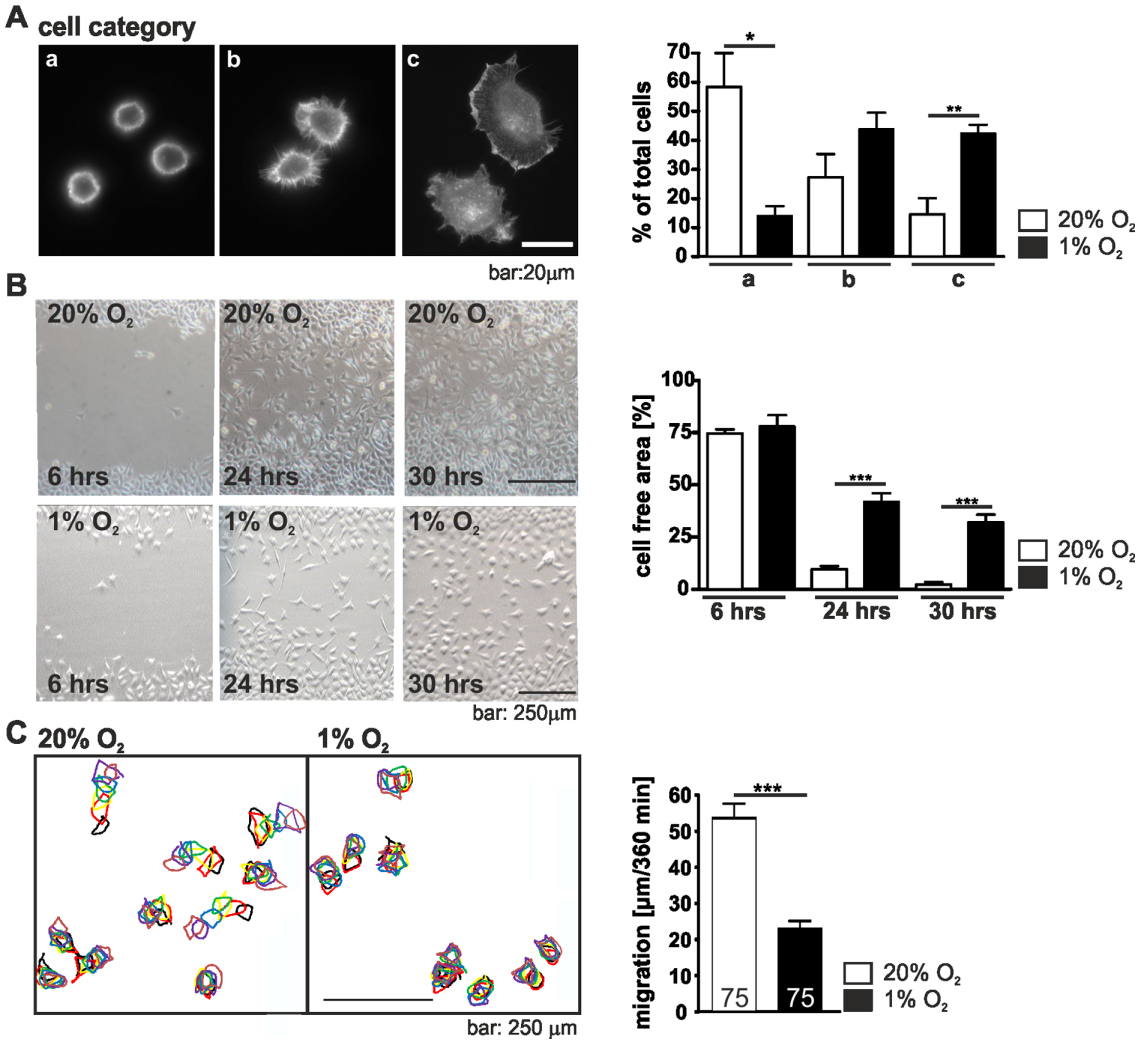
Hypoxia changes cell spreading and migration. (A) Spreading of L929 cells in normoxia and hypoxia. L929 cells were incubated under normoxic (20% O_2_) or hypoxic conditions (1% O_2_), trypsinized and replated for 20 min. Cells were fixed, stained with phalloidin-FITC and divided into three categories (a: round, barely spread; b: in the course of spreading; c: well spread). The percentage of cells in each category was determined. Note that L929 fibroblasts spread faster under hypoxic conditions. (B) Scratch wound healing assays under normoxic and hypoxic conditions. The cell monolayer was scratched with a sterile pipette tip. Images were taken 6, 12, 30 hrs after wounding and the cell-free area was determined. Note that L929 fibroblasts in hypoxia migrate slower into the scratch. (C) Single cell migration of L929 fibroblasts. After 24 hrs of normoxic and hypoxic incubation bright field images of the cells were taken over a period of 360 min. Images were superimposed and the path of the cells was reconstructed by monitoring the cells at each timepoint (going from black to brown). Note that single cell migration is slower under hypoxic conditions. Numbers within the bars indicate the number of cells analysed. * *p*<0.05, ** *p*<0.01, *** *p*<0.001. Bars represent mean values±SEM.

As integrins initiate the formation of focal adhesions the consequence of their higher number in hypoxia on the early stages of cell attachment and spreading behaviour was analysed. Cells grown under normoxic or hypoxic conditions were suspended, replated, cultured and allowed to attach for 20 min, then fixed and stained with phalloidin-FITC. Cells were grouped into three categories: category A, round cells, weakly adhered; category B, weakly adhered cells in the course of spreading; category C, flat cells, well spread. Examples of each category are shown in Figure 2A. Compared with cells grown under normoxic conditions hypoxia was found to considerably enhance early cell spreading of L929 cells and notably more hypoxic cells were found in the group of flat, well spread cells whereas the normoxic cells were barely spread.

The observed changes in early cell spreading prompted us to investigate the influence of hypoxia on L929 cell migration as both processes depend on the cell attachment. A L929 monolayer was scratched with a sterile pipet tip and wound closure was analysed. We observed a marked reduction in the overall speed of wound closure in hypoxic cells. Whereas the normoxic L929 cells almost closed the wound within 30 hrs, the hypoxic cells were markedly delayed (Fig. 2B). Cell proliferation of L929 cells in hypoxia was hold up compared to normoxic conditions. In normoxia L929 cells had a doubling time of 10.69±0.03783 hrs whereas in hypoxia the doubling time was 12.17±0.1974 hrs. Thus, to further validate the hypoxic effect on cell migration a single cell motility assay was performed. For this purpose cells were seeded at very low density to minimize the effect of cell-cell contacts and individual moving cells, excluding those undergoing mitosis, were analysed over a 360 min period under normoxic and hypoxic conditions. Consistent with our findings from the scratch assay hypoxic cells displayed a significant reduction in single cell random migration (Fig. 2C). In summary, our results from both, the scratch assay and the single cell migration assay clearly show that hypoxia causes decreased cell migration of L929 cells.

### Hypoxia Changes the Organization of Cytoplasmic Actins

**Figure 3:**
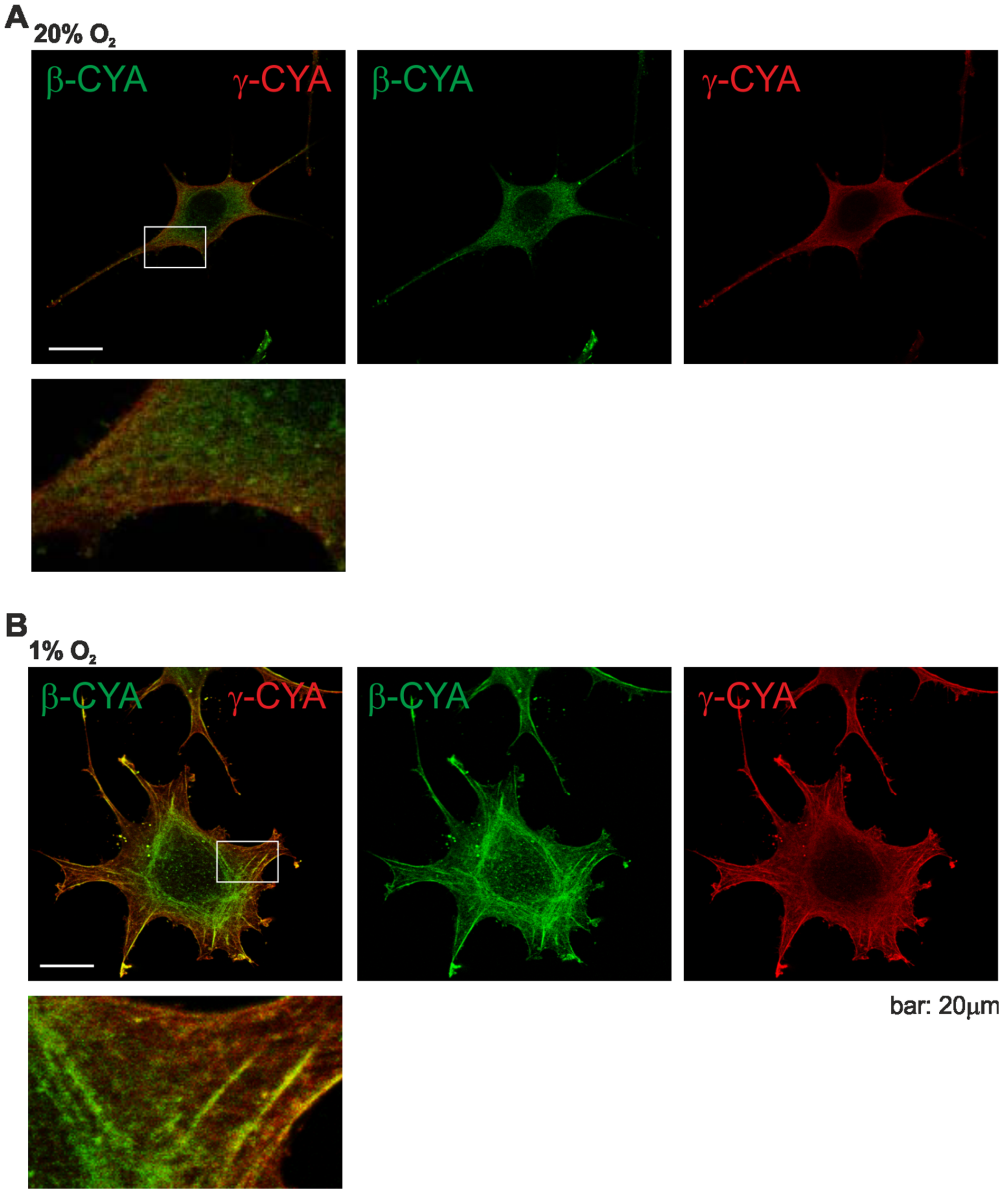
Hypoxia induces the reorganisation of the actin cytoskeleton. β- and γ-actin distribution in L929 fibroblasts in normoxia (A) and in hypoxia (B). Cells were grown for 24 hrs in normoxic or hypoxic conditions and subsequently stained for β-actin and γ-actin. Under normoxic conditions both actin isoforms colocalize. In contrast, under hypoxic conditions the cells develop ring-like actin bundles in the cell center, which are greatly enriched with β-actin, while γ-actin is enriched at the cell periphery.

As cell spreading and cell migration depend on actin polymerization and actin networks, we determined the actin stress fiber complement of L929 cells kept in both conditions, and analysed the subcellular distribution of the β- and γ-actin isoforms. Using highly specific antibodies [21], immunofluorescence staining and confocal microscopy we found the expected, almost uniform distribution of β- and γ-cytoplasmic actin and a partial overlap of both actin isoforms in L929 cells under normoxic conditions (Fig. 3A). However, this subcellular pattern changed in hypoxia (Fig. 3B). There was a partial segregation of β- and γ- cytoplasmic actin between different forms of F-actin bundles. In hypoxia γ-cytoplasmic actin was preferentially located within fibres at the periphery of the cell whereas in many cells β-cytoplasmic actin accumulated in circular bundles. A differential distribution of both actin isoforms is in accordance with previous reports describing the localization pattern of cytoplasmic β- and γ-actin in stationary and moving cells [21]. However, it was never reported before that hypoxia induces the actin isoform redistribution.

### The Hypoxia-inducible Factor-1α Regulates Cell Area Increase Under Hypoxia

**Figure 4:**
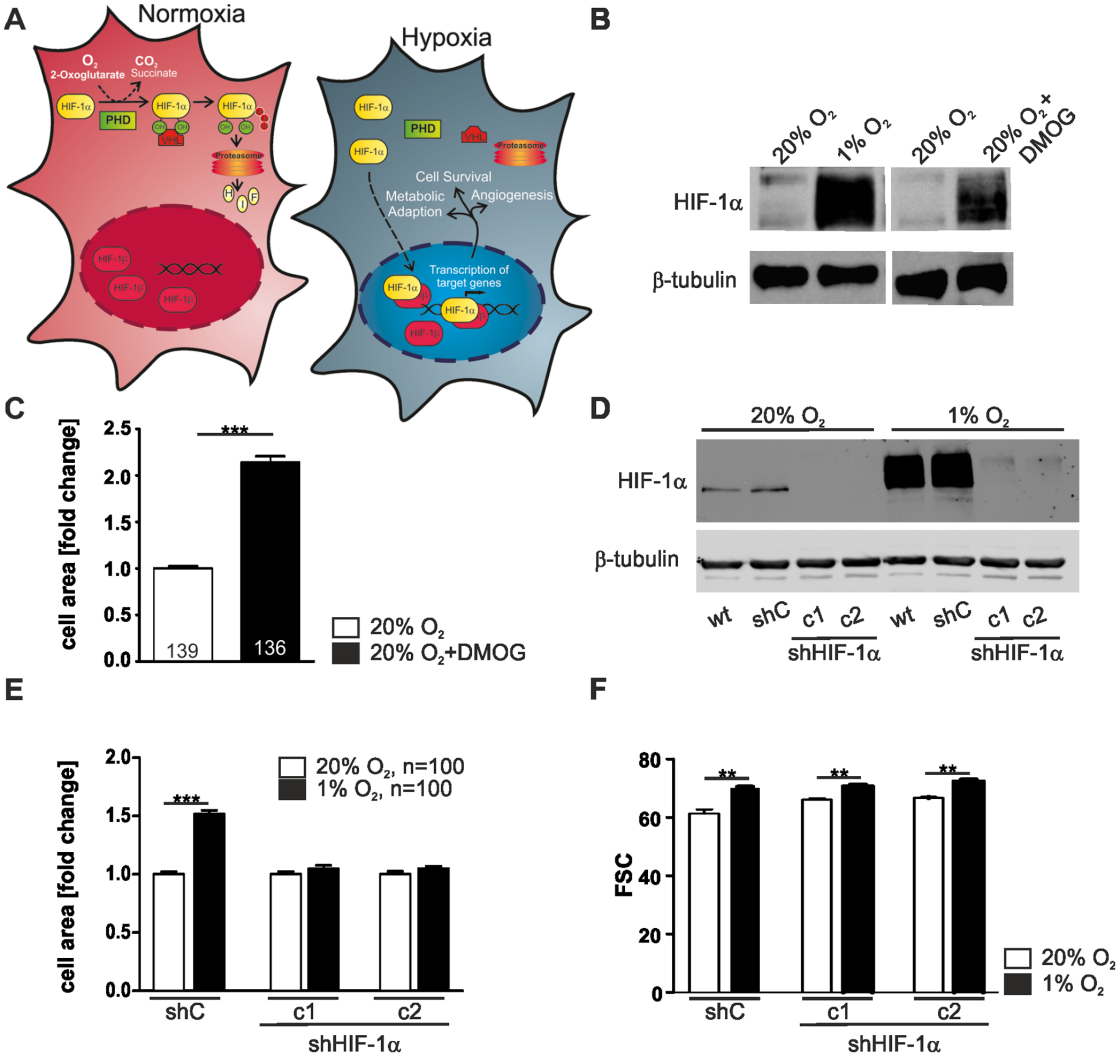
HIF-1α stabilisation regulates cell area. (A) Schematic drawing of HIF-1 and its regulation in normoxia and hypoxia. The stability of the HIF-1α subunit is regulated by prolyl-4-hydroxylase domain (PHD) enzymes in an oxygen dependent manner. Following hydroxylation of the critical prolyl residues under normoxic conditions, the ubiquitin ligase von Hippel-Lindau tumor suppressor protein (pVHL) recognises HIF-1α subunits and targets them for rapid ubiquitination and proteasomal degradation under normoxic conditions. Hypoxia impairs the hydroxylation, which results in HIF-1α stabilisation, nuclear accumulation, heterodimerisation with HIF-1β and subsequent hypoxia-inducible gene expression. (B) Inhibition of PHDs by DMOG causes HIF-1α stabilisation under normoxic conditions. L929 cells were treated with 1 mM DMOG for 48 hrs and total extracts were analysed by immunoblot with the respective antibodies. (C) The cell area of DMOG treated cells increased significantly. The cell area of single cells was measured and was calculated as fold change compared to 20% O_2._ (D) Two HIF-1α knock down cell clones (c1, c2) and a non-target control shRNA cell clone (shC) were obtained via stable transduction of specific sh-plasmids. The HIF-1α knock down was verified in western blot experiments of cell lysates from cells incubated at 20% O_2_ or 1% O_2_. β-tubulin was used as a loading control. (E) HIF-1α knock down cell clones (c1, c2) do not respond to hypoxia with an increase in cell area. The cell area of single cells was measured and was calculated as fold change compared to the cell clone cultivated at 20% O_2_. (F) Flow cytometry analysis of cell volume after incubation in normoxia and hypoxia. shC, c1 and c2 cells were harvested after 24 hrs of normoxic (20% O_2_) or hypoxic (1% O_2_) incubation. Single cell suspension was prepared by enzymatic digestion. Note that hypoxia increases the cell volume independently of the HIF-1α knock down.

HIF-1 is the main transcription factor involved in the cellular response to decreased oxygen availability [22] (Fig. 4A). It is a heterodimeric complex consisting of the stable β-subunit and the α-subunit that is rapidly degraded in normoxia [23]. This degradation is controlled by the prolyl-4-hydroxylase domain enzymes (PHDs) which, in the presence of oxygen, hydroxylate HIF-1α and thus target it for proteasomal degradation [24], [25]. Inhibition of the PHDs results in the stabilisation of HIF-1α under normoxic conditions and the subsequent expression of HIF-1 target genes that are involved in metabolic adaptation, cell survival, angiogenesis etc. [26].

To gain further insight into the interrelationship between HIF-1 and the observed morphological changes in L929 cells we inhibited the enzymatic activity of the PHDs using the oxoglutarate analog dimethyloxalylglycine (DMOG) [27]. As expected we observed a normoxic stabilisation of HIF-1α in DMOG-treated L929 cells, which was comparable to the stabilisation of HIF-1α in hypoxia (Fig. 4B). This was correlated with a significant increase in cell area indicating that this morphological change either depends on PHD-activity or on the stabilisation of HIF-1α (Fig. 4C). To discriminate between these possibilities, we used an shRNA approach and established the HIF-1α L929 knock down cell lines L929 HIF-1α clone 1 (c1) and L929 HIF-1α clone 2 (c2) (Fig. 4D). Simultaneously L929 cells that stably express a non-target control shRNA (shC) were generated to serve as controls. Under hypoxic conditions L929 cells and shC cells showed a strong stabilisation of HIF-1α. This effect was greatly reduced in both HIF-1α knock down cell clones c1 and c2 as seen in Western Blot analysis (Fig. 4D). Hypoxia caused an increase in cell area in the shC cells (Fig. 4E). Remarkably, this increase in cell area was completely abolished in both of the HIF-1α knock down cell clones (Fig. 4E). However in flow cytometry the cells still showed a rightward shift of the forward-angle light scatter indicating an increase of cell volume (Fig. 4F). In summary, our data indicate that the change in cell area in hypoxia is HIF-1α dependent, while the change in cell volume is not regulated by HIF-1α.

### Focal Contact Formation, Enhanced Spreading and Sheet Migration of L929 Cells in Hypoxia is Independent of HIF-1α

**Figure 5:**
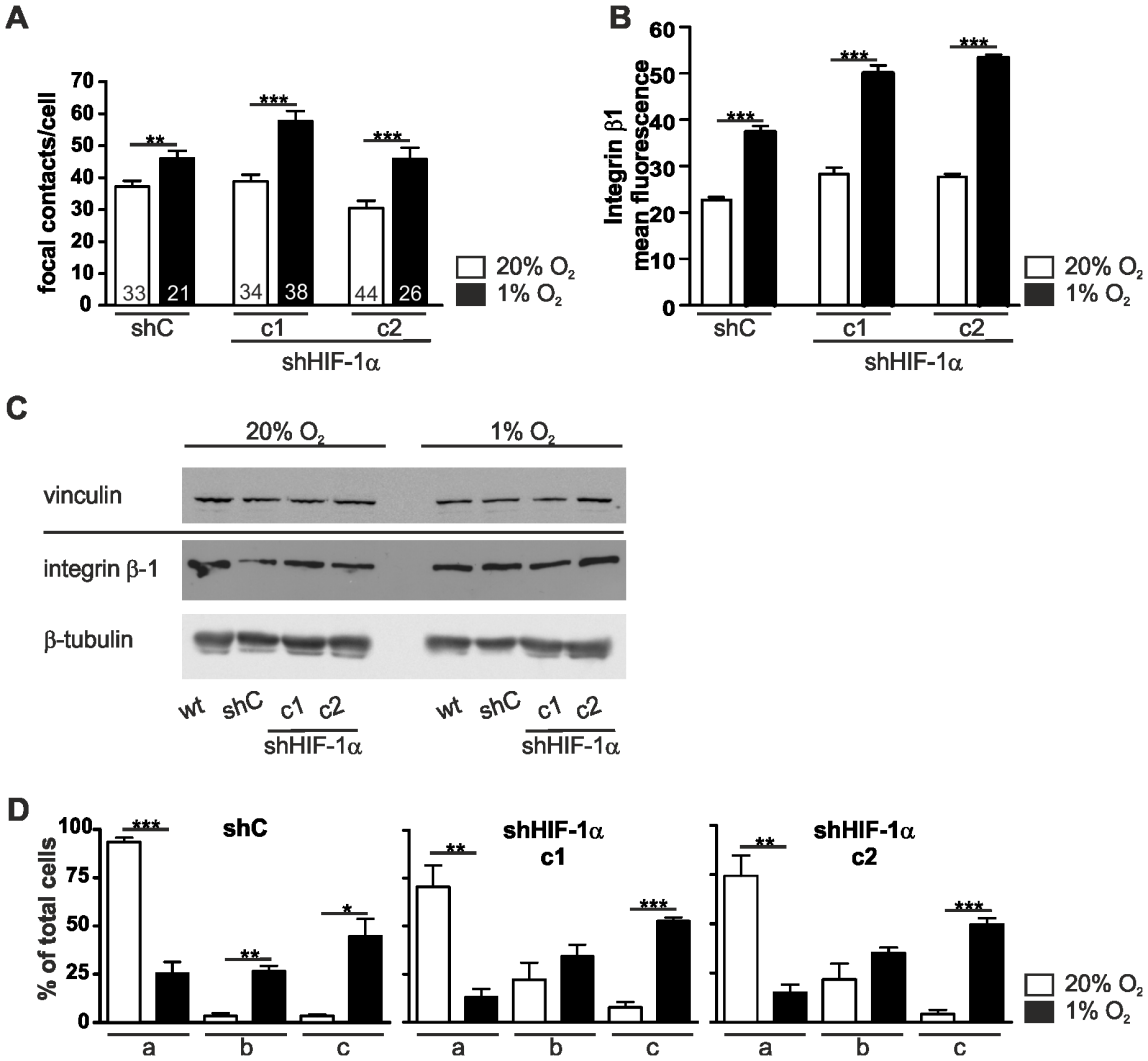
Depletion of HIF-1α does not affect the number of focal contacts and cell spreading. (A) Two HIF-1α knock down cell clones (c1, c2) and a non-target control shRNA cell clone (shC) were stained for vinculin 24 hrs after normoxic (20% O_2_) or hypoxic (1% O_2_) incubation. Counting vinculin positive focal contacts showed a higher number of focal contacts in hypoxia in all three cell lines. (B) Flow cytometry analysis of the HIF-1α knock down cell clones c1 and c2 and the non-target control shRNA cell clone (shC) after 24 hrs of normoxia or hypoxia stained with integrin β1 antibodies. (C) Wt, shC, and the HIF-1α knock down cell clones c1 and c2 cells were lysed after 24 hrs of normoxia (20% O_2_) or hypoxia (1% O_2_). Cell extracts were analysed by Western blots. Note that vinculin and integrin β1 levels are not changed in hypoxia. (D) Cell spreading of the HIF-1α knock down cell clones c1 and c2 and the non-target control shRNA (shC) cell clone in normoxia and hypoxia. Cells were incubated at 20% O_2_ and 1% O_2_, trypsinised and replated for 20 min. Cells were fixed and stained with phalloidin-FITC and divided into three categories (a: round, barely spread; b: in the course of spreading; c: well spread). The percentage of cells in each category was determined. All three cell lines spread faster under hypoxic conditions. Numbers within the bars indicate the number of cells analysed. ** p<0.01, *** p<0.001. Bars represent mean values±SEM.

Having observed that the hypoxic changes of cell shape are HIF-1α dependent, we were interested in the functional significance of HIF-1α in the context of focal contact formation and cell spreading. Similar to the wild type (wt) L929 cells the number of vinculin-positive focal contacts increased in hypoxia in shC cells (Fig. 5A). A comparable effect was also seen in both of the HIF-1α knock down cell clones c1 and c2. This increase in focal contacts in the control and knock down cell clones was corroborated by flow cytometry data which show an increase in β1-integrin at the cell surface (Fig. 5B). However, total vinculin and β1-integrin protein levels were not changed in hypoxia compared to normoxia (Fig. 5C). Furthermore, we examined the spreading of L929 cells in the absence of HIF-1α (Fig. 5D) and did not find any marked changes in the spreading capability. In wt, shC and the HIF-1α knock down cell clones the spreading kinetics increased in hypoxia.

**Figure 6:**
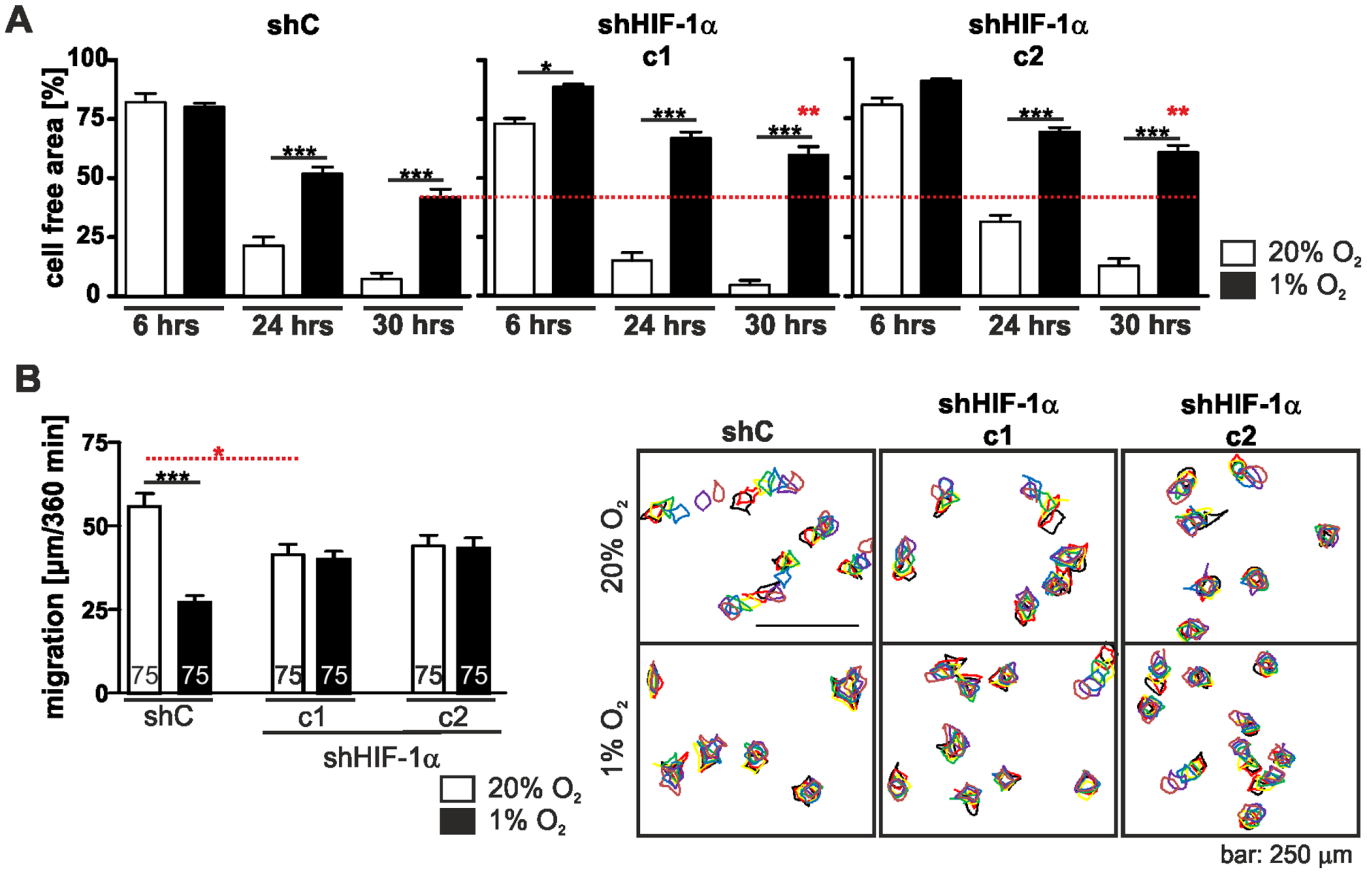
Depletion of HIF-1α alters cell motility. (A) Wounding assay of the HIF-1α knock down cell clones c1 and c2 and the non-target control shRNA (shC) cell clone in normoxia and hypoxia. Cells were grown in normoxia and hypoxia and experimental wounds were caused by scratching cell monolayers with a pipet tip. Images were taken at the indicated time points and the cell free area was determined. Hypoxia delayed wound healing. Note that knocking down HIF-1α slowed wound closure down even more (dashed red line). (B) Single cell migration of the HIF-1α knock down cell clones c1 and c2 and the shC cells. Cells were incubated at 20% O_2_ and 1% O_2_ for 24 hrs. Images were taken over a time period of 360 min and cell movement was analysed. shC cells showed a reduced migration under hypoxic condition, however this effect was not seen in the HIF-1α knock down clones c1 and c2. Numbers within the bars indicate the number of cells analysed. ** p<0.01, *** p<0.001. Bars represent mean values±SEM.

In cell wounding assays the migration behaviour of shHIF-1α c1 and shHIF-1α c2 resembled that of shC cells under normoxic conditions (Fig. 6A). The proliferation of all three cell lines was also comparable (generation time shC: 11.62±0.1458 hrs; generation time shHIF-1α c1∶11.13±0.2628 hrs; generation time shHIF-1α c2∶10.92±0.4984 hrs). However, under hypoxic conditions wound closure in the HIF-1α knock down clones c1 and c2 was even more delayed than in the shC cells (Fig. 6A). The generation times were prolonged compared to normoxic conditions but were comparable between cell types (generation time shC: 14.41±0.03036 hrs; generation time shHIF-1α c1∶13.25±0.06483 hrs; generation time shHIF-1α c2∶13.27±0.2580 hrs).

### Single Cell Migration and Actin Remodelling of L929 Cells in Hypoxia Depend on HIF-1α

In single cell migration experiments both HIF-1α shRNA clones c1 and c2 showed a slight decrease in migration under normoxic conditions when compared to the shRNA control clone. However, hypoxia did not change the migration of the HIF-1α knock down clones whereas the migration of shC cells was significantly reduced (Fig. 6B). These results point to a different role of HIF-1α in sheet migration as seen in wounding assays and single cell migration under hypoxic conditions.

**Figure 7:**
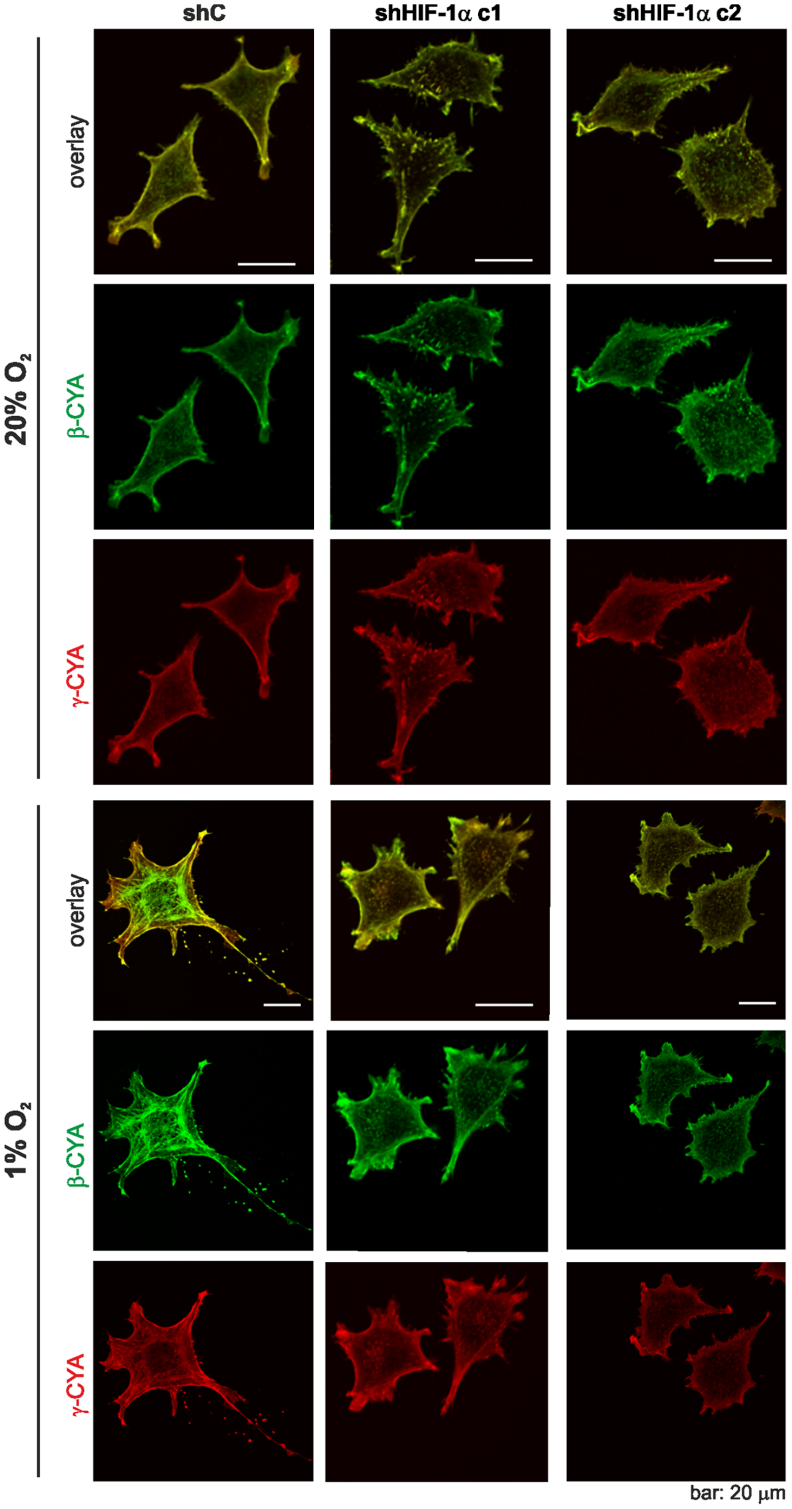
HIF-1α knock down affects cytoplasmic actin reorganisation in hypoxia. ShC cells, and the HIF-1α knock down cell clones c1 and c2 cells were incubated at 20% and 1% O_2_ for 24 hrs and stained for β-actin and γ-actin. Whereas the shC cells display an actin isoform redistribution in hypoxia no obvious actin reorganisation is seen in c1 and c2 cells.

Remarkably, when we analysed the distribution of cytoplasmic β- and γ-actins in the HIF-1α knock down L929 cell clones we did not see differences between cells under hypoxic or normoxic conditions. The shC cells showed a redistribution of β- and γ-actins in hypoxia similar to wt L929 cells (Fig. 7).

### Cofilin Phosphorylation Depends on HIF-1α Expression

**Figure 8:**
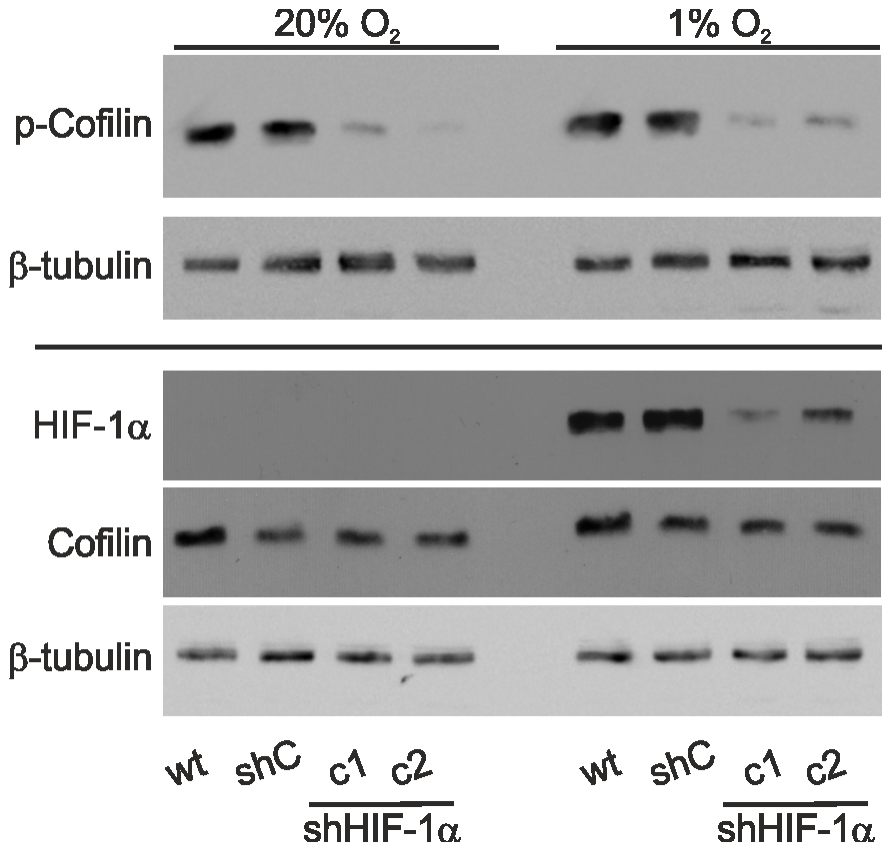
Cofilin phosphorylation is HIF-1α dependent. Wt, shC, and the HIF-1α knock down cell clones c1 and c2 cells were lysed after 24 hrs of normoxia (20% O_2_) or hypoxia (1% O_2_). Cell extracts were analysed by Western blots. Note that p-cofilin levels are reduced in c2 and c2 cells compared to wt and shC cells at 20% O_2_ and 1% O_2_.

Actin dynamics are modulated by cofilin, an actin-binding and severing protein. Cofilin activity is regulated via phosphorylation. LIM kinase dependent phosphorylation inactivates cofilin and thus promotes F-actin assembly, whereas dephosphorylated cofilin is active. This results in severing of actin filaments and sequestering of actin monomers from the pointed end of the filament [28]. Elevated cofilin phosphorylation was observed in hypoxic cells [29], [30] and tissues [31]. This led us to investigate the expression and phosphorylation status of cofilin in L929 cells by Western blot. In wt and shC cells the phosphorylation of cofilin was slightly elevated in hypoxia (Fig. 8). In the HIF-1α knock down cells we noted a marked reduction in cofilin phosphorylation as compared to wt and shC cells. This decrease was seen under normoxic and hypoxic conditions with no obvious changes in total cofilin protein levels. Hence, these data provide evidence for a direct link between HIF-1α expression and cofilin phosphorylation that may be pivotal for the hypoxia induced changes in L929 fibroblast morphology and function as described above.

## Discussion

**Figure 9:**
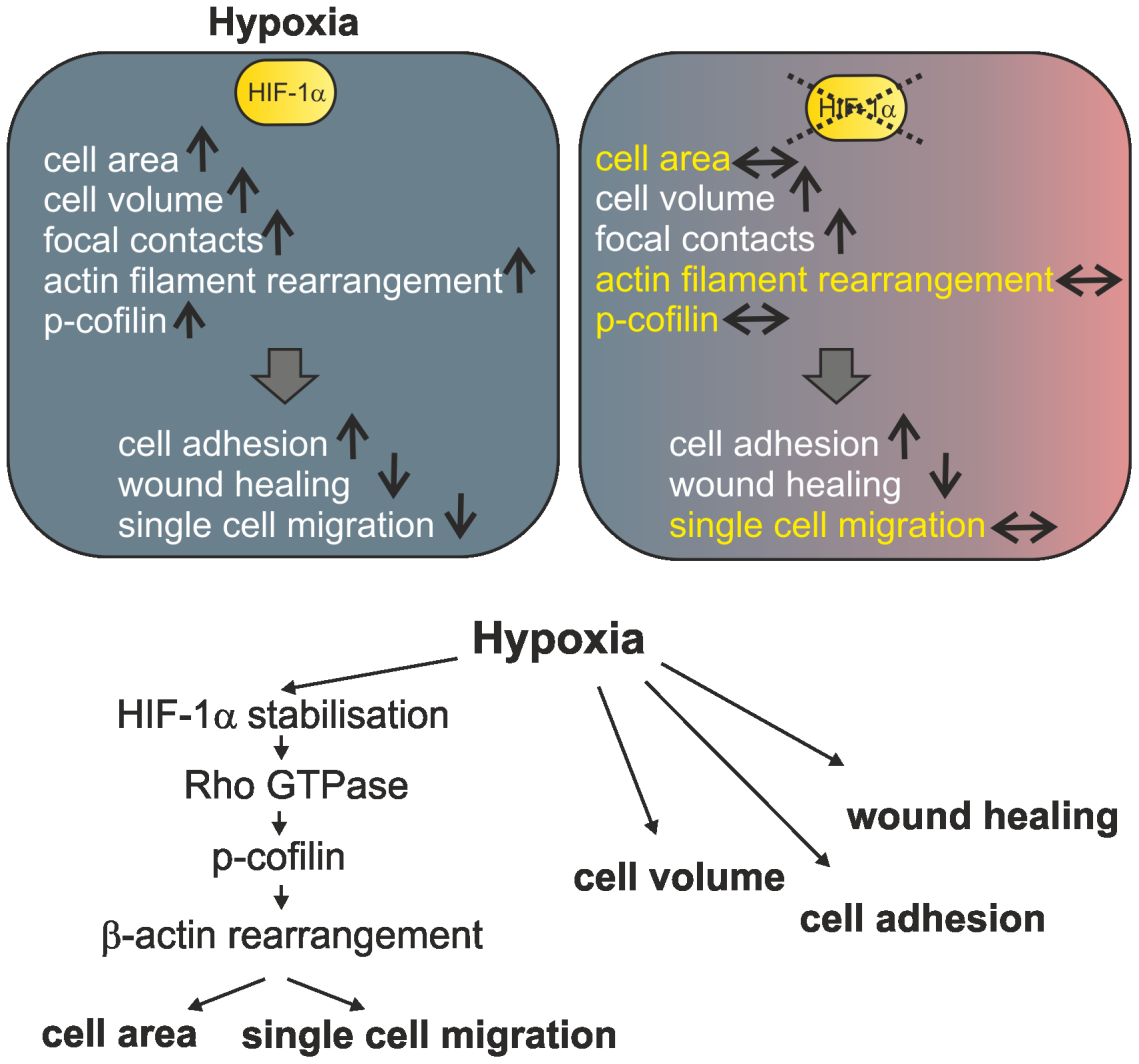
Schematic summary of hypoxia induced and HIF-1α dependent changes in L929 fibroblasts. Hypoxia induces changes in cell architecture and function. Some of these changes can be linked to stabilisation of HIF-1α.↑, increase; ↓ decrease; ↔, no change when compared to normoxia. HIF-1α dependent effects, which are not seen in hypoxia after HIF-1α knock down, are highlighted in yellow.

Fibroblasts encounter hypoxic environments in physiological and pathological conditions e.g. development, cardiovascular diseases and wound healing. However little is known about the morphological and functional consequences of hypoxia on fibroblasts so far. In the current study, we demonstrate that hypoxia strikingly changes cell area, cell volume, cell adhesion and motility in L929 fibroblasts. With the exception of the changes in cell volume and adhesion these phenomena can be accounted for by the stabilisation of HIF-1α. We can link HIF-1α stabilisation to p-cofilin levels with a rearrangement of cytoplasmic β-actin and changes in L929 cell morphology and function (Fig. 9).

Our studies show an increase in L929 fibroblasts cell volume in hypoxia. This increase is independent of HIF-1α stabilisation as it was also seen after knock down of HIF-1α. Increases in cell volume of cerebral microvascular endothelial cell monolayers and of astrocytes in hypoxic conditions have been reported in previous studies [32], [33] and have been found to be partially dependent on Na-K-Cl cotransporter and Na/H exchange activity. Parallel to an increase in cell volume we noticed an increase in cell area of L929 fibroblasts under hypoxic conditions. As hypoxia is known to induce changes in the cytoskeleton, e.g. the exposure of endothelial cells to hypoxia causes a shift in filamentous actin from a web like structure to parallel stress fibres [34], we speculated that a change in cell morphology might depend on cytoplasmic actin rearrangement. Monitoring the distribution of actin isoforms we found that under normoxic conditions both β- and γ-cytoplasmic actin partly colocalised, while hypoxia provoked a reorganization of the cytoplasmic actin network with enhanced formation of β-cytoplasmic actin containing stress fibres around the cell centre and with γ-cytoplasmic actin enriched in a meshwork at the periphery of the cell. As the intrinsic polarity of the cytoskeletal components is required for directed movement, these findings suggest that the formation of circular β-cytoplasmic actin bundles may be causative for the reduced single cell migration of L929 cells in hypoxia. The cytoplasmic actin rearrangement was dependent on HIF-1α stabilisation and was not observed in shHIF-1α knockdown L929 cells. It is coherent that shHIF-1α knock down fibroblasts did not show an increase in cell area and a further reduction in single cell migration in hypoxia.

In hypoxia we also observed the accumulation of integrins and vinculin containing focal contacts. The most obvious roles for focal contacts involve their ability to dynamically connect the cell membrane with the substratum and thus allow cells to attach, detach and move. As a consequence of hypoxia cell spreading of L929 cells was altered and significantly faster compared to normoxic conditions. This is in line with the observation that the accumulation of β1-integrin at the cell surface of HeLa cells results in more efficient cell spreading [35] and that increasing vinculin expression in 3T3 cells also increased the formation of focal contacts and enhanced cell spreading [36]. On the other hand, an increase in vinculin containing focal contacts has been shown to suppress cell motility in wound closure experiments and to reduce individual cell locomotion [36]. This finding provides another possible explanation for the observed effect of hypoxia on cell migration. HIF-1α depletion did not influence β1-integrin accumulation or focal contact number in hypoxia. Neither did it reverse the hypoxic cell adhesion phenotype implying that these are at least in part HIF-1α independent effects. However the impaired wound healing under hypoxic conditions was even further reduced after HIF-1α knock down. Taken together our data show that HIF-1α is involved in L929 cell migration in wound healing experiments as well as in single cell migration. Nevertheless, depending on the migration mode (single cell migration versus “sheet migration” in wound healing experiments), the underlying mechanisms seem to differ and it is feasible that multiple pathways are involved.

The dynamic control of the actin cytoskeleton is essential for cell migration and is mediated by RhoGTPases which control a multitude of actin-binding proteins [17], [37]. For instance Rho-ROCK and Rac-PAK signalling pathways activate LIM-kinase that in turn can phosphorylate and inhibit the actin filament severing protein cofilin [28]. This results in a reorganisation of the actin cytoskeleton. The importance of cofilin in remodeling processes and the maintenance of a polarised cytoskeleton, which is a prerequisite for directional cell migration, has been shown in several studies and the phosphorylation and inactivation of cofilin are necessary for motility to occur [38], [39]. In stably transfected HIF-1α knock down L929 cells we noticed a massive decrease in p-cofilin levels with no obvious change in total cofilin. Consistent with the assumption that cofilin phosphorylation and dephosphorylation reactions control actin dynamics and are important for actin-based motility [40], cell migration was impaired in a monolayer wounding assay in hypoxia and single cell migration assays in normoxia. Hypoxic inhibition of fibroblast migration may contribute to the regulation of fibroblasts in wound healing as the accumulation of fibroblasts is an important factor of tissue repair after injury.

As the reorganisation of the actin cytoskeleton has been associated with a plethora of cellular functions including cell morphology, cell spreading and motility, the mechanisms by which actin dynamics are regulated are diverse. We show that a switch of L929 fibroblasts from normoxia to hypoxia induces changes in the actin cytoskeleton, mainly the redistribution of β-cytoplasmic actin, which goes along with an increase in cell area and volume, enhanced cell spreading and reduced cell locomotion. The increase in cell area and the reduction of migration are related to HIF-1α stabilisation and p-cofilin levels. While cofilin phosphorylation has been linked to hypoxia before, here we show a direct involvement of HIF-1α stabilisation on p-cofilin levels. Taken together, our results bring new insights into the influence of hypoxia on cell function, linking the HIF-signalling pathway to actin dynamics.

## Materials and Methods

### Cell Culture and Treatments

L929 fibrosarcoma cells (ATCC # CCL-1), derived from mouse, were cultivated in high glucose modified Eaglés medium (Pan Biotech, Aidenbach, Germany) containing 10% fetal calf serum (Biochrom, Berlin, Germany), 50 units/ml penicillin G, and 50 µg/ml streptomycin (Pan Biotech). Cells were cultivated in a humidified 5% CO_2_, 95% air atmosphere at 37°C. For hypoxic conditions, O_2_ levels were decreased to 1% with N_2_ in an in vivo 400 work station or SCITIVE work station (Ruskinn, Pencoed, UK). In some experiments cells were treated with 1 mM DMOG (Alexis, Grünberg, Germany).

L929 HIF-1α knock down clones were generated by lentiviral transduction with the pLKO.1-puro HIF-1α-shRNA expression vector (#TRCN0000003810, Sigma-Aldrich, St. Louis, USA). For generating the shControl (shC) transfected cells, a pLKO.1-puro vector was used containing a non-targeting shRNA (#SHC002, Sigma-Aldrich).

For lentiviral transduction, viral particles were produced in HEK293T cells using the ViralPower lentiviral expression system according to the manufactureŕs instructions (Life Technologies, Paisley, UK). Cells were treated with 20 µg/ml puromycin (Life Technologies) to select the cells with successfully integrated plasmid. Two shHIF-1α subclones (c1 and c2) and one shControl expressing clone were established.

### Cell Proliferation

The growth rates of all cell lines were measured by plating 5×10^4^ cells/well as three biological replicates. On days 1, 2, 3, and 4 after plating, cells were dispersed by trypsin treatment and counted. The experiment was repeated three times.

### Fluorescence Staining

Cells were grown on coverslips. For anti-vinculin (hVin-1, V9264; Sigma, Sigma-Aldrich, Steinheim, Germany) and phalloidin-Alexa Fluor 488 (Life Technologies) staining cells were fixed with 4% paraformaldehyde for 20 min. Subsequently, cells were washed with PBS and incubated with 0.1% Triton X for 15 min. β- and γ-cytoplasmic actin were stained as described in Dugina et al., 2009. The following secondary antibodies were used: Texas red- conjugated goat anti-mouse (Santa Cruz Biotechnology, Heidelberg, Germany), TRITC-conjugated goat anti-mouse IgG2b and FITC-conjugated goat anti-mouse IgG1 (Southern Biochtechnology, Birmingham, AL, USA). Images were acquired using a confocal microscope (Zeiss SP2, Carl Zeiss, Göttingen, Germany) or an inverted microscope (Axio Observer D1, Carl Zeiss, Göttingen, Germany).

### Cell Area

Single cells were analysed by bright field microscopy. Pictures were taken with an Olympus C-5060 Wide Zoom camera and cell area was analysed with ImageJ software.

### Cell Spreading Assay

Cells were grown to 70% confluence. Subsequently cells were trysinized, replated, cultured and allowed to reattach. After 20 min cells were fixed and visualized with phalloidin-FITC staining.

### Flow Cytometry Analyses

For flow cytometry analyses 4×10^4^ cells per ml were seeded into 6-wells as three biological replicates. Cells were detached and washed with FACS buffer containing 1.5% fetal calf serum (Biochrom, Berlin, Germany), and 0.1% sodium azide (Carl Roth GmbH+Co. KG, Karlsruhe, Germany) in PBS. Staining was performed with APC-conjugated anti-mouse β1 integrin antibody (HMβ1-1, 102215, BioLegend, London, UK) or IgG Isotype control (HTK888, 400911, BioLegend) for 20 min at 4°C according to manufacturer’s recommendation. Cells were analysed using the BD FACSCanto flow cytometer system (Becton Dickinson GmbH, Heidelberg, Germany), BD FACSDiva and WinMDI2.9 software.

### Single Cell Migration

For single cell migration experiments cells were seeded at 1×10^4^ cells per ml and monitored by a NIKON AZ100 camera. Pictures were taken every 60 min. Single cells were encircled and the pictures were overlaid which allowed us to follow the migration of the cells. Dividing cells were excluded.

### Scratch Assay

For scratch assays, 1×10^5^ cells per ml were seeded into 6-wells as 6 biological replicates. Cells were grown until they reached almost 100% confluence. Using a sterile 200-µl pipette tip, the cell layer was scratched in each well to create a cleared line. The scratch was photographed with an Olympus C-5060 Wide Zoom camera (in normoxia) or with a NIKON AZ100 microscope (in hypoxia) at different time points and the cell-free area was determined using ImageJ software.

### Protein Extraction and Immunoblot Analysis

Cells were lysed in 400 mM NaCl, 1 mM EDTA, 10 mM Tris/HCl, pH 8.0, and 0.1% TritonX100 including the protease inhibitor cocktail cOmplete Mini (Roche Applied Science, Mannheim, Germany). For p-cofilin and β1-integrin detection cells were lysed in 8 M urea and 1% CHAPS. Subsequently, lysates were treated with ultrasound (SONOPLUS HD 2070, Bandelin GmbH, Berlin, Germany). Protein concentrations were determined by the Bradford method using BSA as a standard. For immunoblot analysis, protein extracts were electrophoresed through sodium dodecyl sulfate (SDS)-polyacrylamide gels and electro-transferred onto nitrocellulose membranes (Amersham, Freiburg, Germany) by semi-dry blotting (PeqLab, Erlangen, Germany). For detection of specific proteins, the following primary and secondary antibodies were used: anti-HIF-1α (NB100-479; Novus, Littleton, USA), anti-β-tubulin (Abcam, Cambridge, UK, ab6046), anti-cofilin (Abcam, ab42824), anti-p-cofilin (Santa Cruz Biotechnology, Heidelberg, Germany, sc-12912-R), anti-β1-integrin (ab 5297, Abcam), anti-vinculin (hVin, Sigma-Aldrich), horseradish peroxidase (HRP)-labelled anti-mouse (Sc-2005, Santa Cruz) and HRP-labelled anti-rabbit (Sc-2004, Santa Cruz). Chemiluminescence detection of HRP was performed by incubation of the membranes with 100 mM Tris-HCl, pH 8.5, 2.65 mM H_2_O_2_, 0.45 mM luminol, and 0.625 mM coumaric acid for 1 min followed by imaging using the LAS3000 system (Fuji) or Amersham Hyperfilm ECL (GE Healthcare, Muenchen, Germany) autoradiography films.

### Statistical Analyses

Results are expressed as mean±SEM. For statistical comparisons, results were analysed by unpaired Students *t-*test (Figs 1, 2; Fig. 4C, E, F Fig. 5A, B, C) and one-way ANOVA (Fig. 5D, F) followed by Bonferroni *post hoc* tests. Values of p<0.05 (*), p<0.01 (**), p<0.001 (***) were considered statistically significant.
